# The analgesic effect of green light on neuropathic pain: a mini-review of the literature and a proposal for future work

**DOI:** 10.3389/fpain.2025.1653186

**Published:** 2025-09-03

**Authors:** Wenjing Dai, Ying Zhang, Rui Gu, Xiaoyan Zhu, Yujie Leng, Lijie Ma, Ming Zhang

**Affiliations:** ^1^ Clinical Medical College, Chengdu Medical College, Chengdu, China; ^2^ Pulmonary and Critical Care Medicine, The General Hospital of Western Theater Command, Chengdu, China; ^3^ Department of Cardiovascular Surgery, Xi’an No. 1 Hospital, Xi’an, China; ^4^ Basic Medicine College, Ya’an Polytechnic College, Ya’an, China; ^5^ Department of Basic Medical Laboratory, The General Hospital of Western Theater Command, Chengdu, China; ^6^ Department of Anesthesiology, The General Hospital of Western Theater Command, Chengdu, China

**Keywords:** neuropathic pain, green light, ventral lateral geniculate nucleus, analgesic effect, neuronal plasticity

## Abstract

Medically refractory, severe, and unrelenting neuropathic pain remains a public health challenge worldwide. Green light has been found to have an analgesic effect on neuropathic pain. Interestingly, this analgesic effect is prolonged even after green light exposure. Peripheral and central mechanisms include the inhibition of the inflammatory response and the activation of the endogenous cannabinoid system and nerve circuits between the lateral geniculate nucleus and other brain regions, such as the dorsal raphe nucleus and the rostral ventromedial medulla, which may mediate the analgesic effect of green light. An increasing number of clinical studies highlight the side effects of traditional analgesics. The antinociceptive effect of green light has been proven in fibromyalgia and migraine patients. However, the effect of green light on neuropathic pain has not been reported in clinical settings. Here, we review the cellular and molecular mechanisms of the antinociceptive effect of green light. Furthermore, the green light parameters (intensity, duration, and wavelength) used in clinical trials are also summarized.

## 1 Introduction

Chronic pain has become a global problem. China has the highest number of chronic pain patients, with more than 300 million chronic pain sufferers accounting for an economic cost of at least 60 billion CNY per year (1). Tricyclic antidepressants, nonsteroidal anti-inflammatory drugs, and other painkillers are currently used to treat pain. However, adverse reactions such as sedation, cardiotoxicity, ataxia, addiction, and respiratory depression interfere with treatment (2, 3). Medical organizations, such as the Chinese Orthopaedic Association and the American Academy of Orthopaedic Surgeons, and evidence-based clinical practice guidelines have clearly shown that drug and surgery use is limited by indications, comorbidities, and adverse events. Non-pharmacological interventions are therefore advocated as first-line treatments (4). The analgesic effects of non-pharmacological therapies, including psychological, behavioral, meditative, and physical therapies, have been demonstrated (5). Recently, the application of phototherapy as a treatment for neuropathic pain has become particularly attractive due to its favorable properties, such as low cost and few adverse effects. Different wavelengths, intensities, and routes of light administration have different effects on neuropathic pain. The promising analgesic effects of green light in animal studies have encouraged its consideration for the clinical treatment of neuropathic pain. However, the underlying mechanisms have not been fully clarified. This paper focuses on explaining the antinociceptive effect of green light and the biological mechanisms by which it acts on neuropathic pain.

## 2 Effect of green light on physiological pain perception

Owing to the short wavelength of green light, its penetration through the skin may be insufficient to exert analgesic effects on peripheral tissues. To assess the light propagation path, Ibrahim et al. (6) fixed naïve rats with dark, opaque contact lenses. Then, the increase in paw withdrawal thresholds (PWTs) caused by green light was abolished. Thus, the antinociceptive effect of green light depends on the visual transmission system used. Numerous studies have shown that green light can modulate physiological pain perception. As shown in Table 1, green light significantly increased the paw withdrawal latencies (PWLs) of naïve rats (6). Meanwhile, both bilateral PWTs and PWLs were elevated in green light-treated naïve mice (7). Interestingly, PWTs on the left side increased significantly on the 5th day, whereas those of the right side increased on the 7th day (7). The difference in the onset of green light-induced analgesia between the two sides suggested that green light may activate different retino-recipient brain regions. To test whether this analgesic effect of green light is related to light intensity, naïve rats were exposed to different intensities of green light, including 4, 12, 36, 110, and 330 lux. All tested green light intensities caused a time-dependent increase in PWLs (6). In contrast to other intensities, 330 lux produced a suboptimal antinociceptive effect. These findings suggested that the analgesic effect of green light is not maximal at its ideal intensity. However, the maximal response to the antinociceptive effect was observed in the 4-lux green light group (6). The possible mechanisms underlying the differences in light intensity and analgesic effects are explained below.

**Table 1:** Effect of green light on physiological pain perception in naïve animals.

Parameters of green light	Animal	Changes in pain behavior	Ref.
4/12/36/110/330 lux, 8 h/day, 5 days	Rat	1. The PWLs and the PWTs were increased in a time-dependent manner following green light exposure (4/12/36/110 lux). 2. Thermal analgesia was similar among rats, which were exposed to 12-, 36-, and 110-lux intensities. 3. The PWLs were increased in the 330-lux group. There was only a half-maximal response when compared with the 12-, 36-, or 110-lux intensity group. 4. The increase in PWLs induced by the 110-lux green light was maintained for 4 days. 5. The thermal analgesia of the 4-lux green light was maintained for 7 days.	(6)
50 lux, 2 h/day, 7 days	Mouse	Bilateral PWTs were elevated significantly after the green light treatment.	(7)

PWT, paw withdrawal threshold; PWL, paw withdrawal latency.

## 3 Analgesic effect of green light on neuropathic pain

As shown in the Table 2, green light completely inhibited thermal hyperalgesia and strongly reversed mechanical hyperalgesia in rats subjected to spinal nerve ligation (SNL) (6). Wu et al. (7) reported that both bilateral PWTs and PWLs were elevated in models of partial sciatic ligation (PSL) after 7 days of green light treatment. Green light has also shown antinociceptive effects on mice with chronic constriction injury (CCI) (11). The effects of different intensities of green light (50, 200, or 500 lux) were assessed in CCI mice. Antinociceptive effects were observed only in the 200-lux green light group (11). Anxiety associated with neuropathic pain was assessed via conditioned place preference tests. The degree of anxiety in CCI mice was clearly reduced by increasing the amount of time spent in the chamber with green light. These findings suggest that both pain-related behavior and pain-like emotional disorders in neuropathic models can be simultaneously modulated.

**Table 2:** Effect of green light on neuropathic pain behavior.

Pain model	Animal	Parameters of green light	Changes in pain behavior	Ref.
CCI	Mouse	50/200/500 lux, 4 h/day, 7/14 days	1. Both PWTs and PWLs increased obviously in the 200-lux green light group. 2. Both PWTs and PWLs were not influenced by the 50/500-lux green light. 3. The antinociceptive effects were extended for 2 days following the end of the 7-day green light treatment. 4. Analgesic effects lasted longer than 10 days after the 14-day green light treatment.	(8)
HIV	Rat	100 lux, 8 h/day, 6 days	1. Mechanical hyperalgesia was significantly inhibited from the second day following green light. 2. The antinociceptive effect of green light was prolonged for 8 days. 3. Thermal analgesia occurred from the fifth day following green light. 4. Thermal sensitivity was continuously suppressed for 6 days.	(9, 10)
PSL	Mouse	50 lux, 2 h/day, 7 days	1. Bilateral PWTs and PWLs were increased significantly following green light from the third day. 2. The spontaneous pain-related aversive behavior was not affected by the green light. 3. The antinociceptive effect of green light on the mechanical hyperalgesia was prolonged for 6 days in the ipsilateral side, while it persisted for 4 days in the contralateral side.	(7)
SCI	Mouse	100 lux, 8 h/day, 14 days	1. Mechanosensitivity was inhibited after the 14-day green light treatment. 2. The PWLs were increased significantly from the second day following green light exposure.	
SNL	Rat	4 lux, 8 h/day, 5 days	1. The PWLs were increased, and thermal hyperalgesia was completely reversed by green light. 2. The PWTs were elevated after green light application. 3. The thermal analgesia of 4-lux green light was extended for 10 days. 4. The PWTs were elevated significantly for the additional 4 days of 4-lux green light.	(6)

CCI, chronic constriction injury; HIV, human immunodeficiency virus; PSL, partial sciatic ligation; PWL, paw withdrawal latency; PWTs, paw withdrawal thresholds; SCI, spinal cord injury; SNL, spinal nerve ligation.

## 4 Peripheral and central mechanisms of green light-induced analgesic effect

### 4.1 Inhibition of the inflammatory response

The effectiveness of green light in attenuating mechanical/thermal hypersensitivity has been confirmed in SCI mice. In the green light treatment phase, the expression of the anti-inflammatory factor IL-10 in the spinal cord was elevated, while the expression of the proinflammatory factors IL-18/6/1β and TNF-α decreased (12). Thus, the analgesic effect of green light on neuropathic pain relies on reduced spinal inflammation. Proinflammatory factors can be released from activated microglia. This type of chronic neuroinflammation exacerbates neuronal damage and promotes neuropathic pain (13, 14). Expression of CD68, a marker of microglial activation, was significantly reduced after green light application (15). These findings suggest that the reduced neuroinflammation in SCI mice is associated with decreased microglial activity. Chronic neuroinflammation in SCI is also influenced by microglial autophagy (16–18). Restoring microglial autophagy can reduce chronic neuroinflammation and promote neuronal survival (18). Increased neuronal survival and reduced neuroinflammation associated with SCI correspond to restored microglial autophagy following green light exposure (12). Green light can reduce neuropathic pain by downregulating inflammatory markers, restoring microglial autophagy, and promoting neuronal survival in spinal cord tissue.

### 4.2 Activation of the endogenous opioid system

In naïve rats, green light treatment increased β-endorphin and pro-enkephalin levels in the cerebrospinal fluid (CSF), but not in the serum. However, dynorphin levels remain unchanged in the CSF or serum following green light exposure (9, 10). The thermal analgesic effect of green light was reversed after the deletion of either the δ- or μ-opioid receptor. Moreover, intrathecal administration of naloxone, a μ-opioid receptor antagonist, also reversed the antinociceptive effects of green light in naïve rats (6). Both thermal and mechanical hypersensitivity in HIV-related chronic neuropathic pain are reduced by green light. This antinociceptive effect also requires the activation of δ- and μ-opioid receptors in the spinal cord (9, 10). Even the green light-induced analgesic effect was blocked following the subcutaneous administration of naloxone (6). It is highly likely that the antinociceptive effects of green light on physiological pain and neuropathic pain are mediated mainly through the central opioid system rather than the peripheral opioid system. In the spinal cord, opioid receptors mediate synaptic plasticity by inhibiting calcium (Ca^2+^) influx and adenylyl cyclase and then decreasing the glutamate (Glu) release in the presynaptic membrane. Moreover, enhanced chloride (Cl^−^) influx and potassium (K^+^) efflux are involved with opioid receptor-mediated postsynaptic inhibition of pain (19). This opioid receptor-mediated neuronal plasticity in the spinal cord may contribute to the antinociceptive effect of the green light.

The pro-enkephalin-A gene is expressed in the suprachiasmatic nucleus (SCN), which receives direct retinal input and regulates circadian rhythms (20). The release of endogenous opioids may be modulated by the SCN (21). It is speculated that the SCN may be one of the first gateways in green light-induced antinociception. Endogenous opioid-expressing neurons are also present in the rostral ventromedial medulla (RVM) (22). There is a direct connection between the olivary pretectal nucleus (OPN), which receives light signals through the optic tract (optic tract–OPN) (5). The green light-induced upregulation of endogenous opioids may originate from the optic tract–OPN–RVM circuit and then facilitate descending pathways. Whether the analgesic effect of green light is related to these hypotheses warrants further study.

### 4.3 Changes of the endocannabinoid system

The endocannabinoid system comprises endocannabinoids, cannabinoid receptors, and metabolizing enzymes. Under neuropathic pain conditions, alterations in the neuronal endocannabinoid system associated with both pain perception and emotional disorders were discussed in our previous review (23). Hind paw mechanosensitivity in osteoarthritic rats was reversed after only two sessions of green light treatment (24). Following green light exposure, serum levels of the analgesic fatty acid amide N-palmitoyl ethanolamine (PEA) and N-acyl glycines were significantly elevated. In contrast, classic endocannabinoid anandamide (N-arachidonoyl ethanolamine) and 2-acyl-sn-glycerol lipid levels, including classic endocannabinoid 2-arachidonyl glycerol, remained unchanged (24). The antinociceptive effect of green light treatment was reversed by treatment with AM281, a G-protein-coupled receptor-18/cannabinoid-1 receptor antagonist. These results suggest that green light may alleviate neuropathic pain by activating the endocannabinoid system by increasing the content of circulating analgesic endocannabinoids. The endocannabinoid-mediated neuronal plasticity in the central nervous system may be driven by green light under a neuropathic state.

### 4.4 Peripheral and central neuronal plasticity

Previous results suggested that hyperexcitability of dorsal root ganglion (DRG) neurons contributes to neuropathic pain (25). Ca^2+^ influx in ATP-responsive rat DRG neurons is increased in individuals with neuropathic pain (26). The excitability of DRG neurons, along with an increased Ca^2+^ influx in ATP-responsive DRG neurons, is mediated by N-type channels. This kind of DRG neuron Ca^2+^ influx was inhibited in green light-exposed rats (6). Thus, functional changes in ATP signaling in DRG sensory neurons contribute to the analgesic effect of green light. At the peripheral level, sensory neuronal adaptations, due to possible changes in the activities of voltage-gated calcium, may help explain the molecular mechanisms underlying the antinociceptive effect of green light.

**Figure 1:**
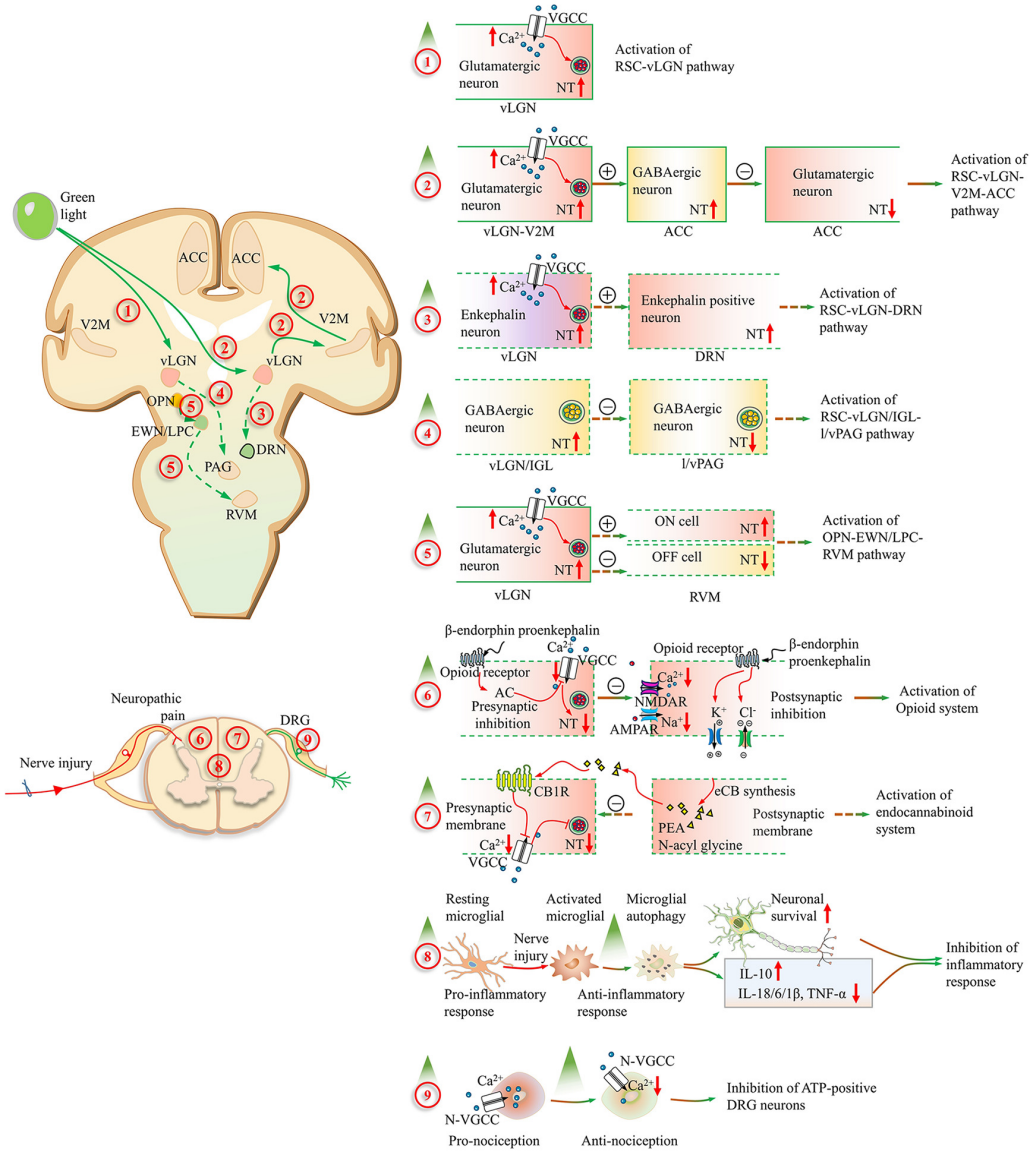
Alterations of the neuropathic pain pathways induced by green light. (1) The excitability of the ventral lateral geniculate nucleus (vLGN) glutamatergic neurons is increased after green light exposure through the retinal ganglion cell (RGC–vLGN^Glu^) pathway. (2) These glutamatergic projections from the secondary visual cortex (V2M^Glu^) to GABAergic neurons in the ACC are activated by green light. Then, local glutamatergic neuron activity is inhibited by GABAergic neurons in the ACC (V2M^Glu^–ACC^GABA−Glu^). (3) The release amount of enkephalin in the vLGN is increased through RGCs and transmitted to the dorsal raphe nucleus. The activation of the RGC–vLGN^ENK^–DRN pathway is involved in the analgesic effect of green light in inflammatory pain. (4) The bright light could activate GABAergic neurons in the vLGN/IGL through RGCs. Nocifensive behaviors are suppressed corresponding to the disinhibition of lateral and ventrolateral parts of the periaqueductal gray area (l/vlPAG) via the activation of the RGC–vLGN/IGL^GABA^–l/vlPAG^GABA^ circuit. (5) The pain could be aggravated following the activation of RVM ON cells and the suppression of OFF-cell firing through the olivary pretectal nucleus (OPN)–Edinger–Westphal nucleus and lateral parabrachial complex (EWN/LPC)–rostral ventromedial medulla (RVM) pathway after the great intensity of bright light exposure. (6) In the spinal cord, the presynaptic inhibition is mediated by the opioid receptor-mediated synaptic plasticity with inhibition of calcium (Ca^2+^) influx and adenylyl cyclase. And then the glutamate (Glu) release is decreased. Moreover, enhanced chloride (Cl^−^) influx and potassium (K^+^) efflux are involved with the opioid receptor-mediated postsynaptic inhibition. (7) The analgesic fatty acid amide PEA and N-acyl glycines are significantly elevated in the serum after green light exposure and inhibit pain by activating the cannabinoid receptor 1. (8) The neuronal survival improvement and neuroinflammation reduction corresponding to the restored microglial autophagy, increased IL-10, decreased IL-18/6/1β, and TNF-α. (9) The analgesic effect of green light is mediated by the reduction of Ca^2+^ influx in ATP-responding dorsal root ganglion neurons, which goes through the N-type channel. A red upward arrow represents an increase, while a red downward arrow represents a decrease. Contents in solid boxes represent facts that have been confirmed by public research. Contents in dashed boxes are unproven inferences or hypothetical things. A plus sign in the circle represents enhancement, and a minus sign in the circle represents reduction. AMPAR, α-amino-3-hydroxy-5-methyl-4-isoxazolpropionic acid receptor; IL-1β, interleukin-1 beta; IL-6, interleukin-6; IL-10, interleukin-10; IL-18, interleukin-18; NMDAR, N-methyl-D-aspartate receptor; NT, neurotransmitter release; TNF-α, tumor necrosis factor-alpha; VGCC, voltage-gated calcium channel.

At the central level, the retinal ganglion cell–lateral geniculate nucleus circuit (RSC–LGN) serves as a gateway for the green light-induced analgesic effect. Wu et al. (7) demonstrated that green light increased bilateral PWTs and PWLs in PSL mice mainly via the activation of glutamatergic neurons in the vLGN (vLGN^Glu^). In contrast, red light exacerbates neuropathic pain through the activation of GABAergic neurons in the vLGN (vLGN^GABA^). There was a great increase in the Ca^2+^ current of glutamatergic neurons in the vLGN in both naïve and PSL mice after green light exposure. Thus, the activation of vLGN^Glu^ neurons in the retino-vLGN circuit may be a primary target for green light-induced analgesia. As shown in Figure 1, the vLGN is an anterior thalamic nucleus that receives projections from retinal inputs and relays information to the visual cortex and other brain regions (27).

The anterior cingulate cortex (ACC) is an important site for processing and regulating neuropathic pain. Pyramid neurons in the ACC exhibit hyperactivity and synaptic plasticity in the context of neuropathic pain (11). This type of activation of the ACC is also involved in the generation of affective components including aversion, anxiety, and depression (28). Functional imaging studies have shown that green light affects functional connectivity in the ACC (29–31). Green light may pass through the relay site LGN and reach the primary and secondary visual cortices (V1 and V2). V1 transforms the information received from the LGN and distributes it to separate domains in V2 (32). The excitability of glutamatergic neurons in the medial part of the secondary visual cortex (V2M) increases following the green light application (8). V2M^Glu^ neurons have multiple axonal projections within the anterior cingulate cortex (ACC), which plays important roles in processing both sensory and affective aspects of neuropathic pain (33). The excitability of GABAergic neurons in the ACC increased, but that of glutamatergic neurons significantly decreased after the green light exposure (8). Thus, green light activates glutamatergic projections from the V2M (V2M^Glu^) to GABAergic neurons in the ACC, leading to the inhibition of local glutamatergic neurons (V2M^Glu^–ACC^GABA−Glu^). Compared with 50-lux and 100-lux green light, only 200-lux green light inhibited neuropathic pain in CCI mice. These findings suggest that activation of the retinal–LGN–V2M^Glu^–ACC^GABA−Glu^ circuit may have light intensity specificity. Glutamatergic neurons of the V2M also project to the anteromedial thalamic nucleus, zona incerta, superior colliculus, and other brain regions (8). However, whether these projections are involved in the antinociceptive effects of green light remains unclear. Moreover, what specific light intensity can activate these circuits is an interesting issue. This may explain why the 330-lux green light produced only a half-maximal response when compared with the other intensities (6).

Sensorimotor integration, active movement, and sensory information can be modulated and carried by ACC downstream circuits. The projection from the ACC to the dorsolateral and lateral periaqueductal gray (dl/lPAG) is associated with both reflexive and active avoidance behavior (34). Furthermore, the descending pathway, comprising the PAG, rostral ventromedial medulla, dorsal raphe nucleus (DRN), spinal dorsal horn, and other nuclei, acts as an endogenous modulator of neuropathic pain (35). Neuronal activity in the nucleus of the descending pathway may contribute to the antinociceptive effect of green light.

The vLGN is well known to be innervated by different retinal cells. Rods, cones, and melanopsin-expressing intrinsically photosensitive retinal ganglion cells (ipRGCs) are the three types of retinal cells with distinct peak excitation wavelengths and photoresponsive characteristics in the mammalian eye (36, 37). Like red light, bright light can also activate GABAergic neurons in the ventral lateral geniculate nucleus and intergeniculate leaflet (vLGN/IGL) through a subset of retinal ganglion cells (RGCs). Unlike red light, which promotes pain, bright light suppresses nocifensive behaviors in mice corresponding to the disinhibition of the lateral and ventrolateral periaqueductal gray area (l/vlPAG)-mediated descending pain inhibitory pathway via the activation of the RGC–vLGN/IGL^GABA^–l/vlPAG^GABA^ circuit (38). These findings suggest that the antinociceptive effects of bright light depend on the RGC–vLGN/IGL^GABA^–l/vlPAG^GABA^ circuit. Whether this circuit may be activated under green light is worth studying.

We also noted that low-intensity green light reduces headache pain scores, whereas higher-intensity green light (100 cd/m^2^) exacerbates migraine (39). Thermal hyperalgesia in naïve rats is also caused by high-intensity bright light (18 × 10^3^ lux) (40). This pronociceptive state results from the light-induced activation of RVM ON cells and the suppression of OFF-cell firing (40). The OPN, a relay in the pupillary light reflex, projects to the RVM through the Edinger–Westphal nucleus and lateral parabrachial complex (EWN/LPC) (41, 42). However, whether the OPN–EWN/LPC–RVM circuit mediates the pronociceptive effect of high-intensity green light remains unclear. The RVM also projects directly or indirectly to the spinal or medullary dorsal horns to modulate nociceptive trafficking (43). The thermal analgesic effect of green light was diminished after the microinjection of lidocaine (6). Moreover, 65 proteins associated with a decreased “structural molecule activity” and increased “antioxidant activity” in DRGs from naïve rats contributed to green light-induced thermal analgesia (6). This result suggested that the activation of the RVM–SC–DRG circuit mediated the green light-induced thermal analgesia. Different subtypes of neurons in the RVM may be associated with pronociceptive effects and antinociceptive effects. This inference requires further research in the context of neuropathic pain.

Tang et al. (44) discovered that the green light-induced antinociceptive effect is conveyed by retinal rod and cone cells but not by ipRGCs. Specifically, the activation of enkephalin neurons (vLGN^ENK^) of the retino-vLGN circuit was shown to contribute to green-induced antinociceptive pain in inflammatory mice. The released enkephalin from the retino-vLGN^ENK^ circuit is subsequently transmitted to the DRN. As a result, the descending pain modulation system originates from the DRN after the interaction between the enkephalin and μ-opioid receptors (44). This study suggested that green light-induced analgesic effects depend on the retinal–vLGN^ENK^–DRN circuit, which includes the visual transmitting pathway and the descending pain inhibitory system. Furthermore, retinal cone cells are essential, rods are necessary, but ipRGCs are not required for green light-induced analgesia. Interestingly, headache pain intensity but not the frequency of headache days was decreased significantly after green light exposure in a colorblind patient; notably, such patients cannot differentiate green, yellow, orange, and red colors (45). This clinical trial suggested that headache pain intensity was modulated by green light through non-image-forming ipRGCs and that headache pain frequency was influenced by the retinal cone and rod cells. These studies suggest that different retinal cells contribute differentially to the analgesic effects of green light. However, whether the analgesic effect of green light on neuropathic pain depends on the retinal–vLGN^ENK^–DRN circuit is unknown. Moreover, the specific types of retinal ganglion cells involved in this circuit should be illuminated in the future.

## 5 The analgesia maintenance of green light

Neuropathic pain behavior was evaluated on subsequent days following the end of green light exposure. Thus, the analgesic effects of the green light, whether transient or prolonged, can be assessed. Ibrahim et al. (6) reported that the increase in the PWLs of naïve rats was maintained for 4 days. An extended analgesic effect of green light under neuropathic pain was also discovered (Table 1). The maintenance of analgesia after the first green light treatment was evaluated. The PWTs of CCI mice increased slightly after 4 h of green light exposure but returned to the initial level within 6.5 h (11). In SNL rats, the antinociceptive effect of green light extends for 10 days in PWLs and 4 days in PWTs (6). Green light-induced antinociception was maintained in the PWTs and PWLs up to day 7 in PSL mice (7). The maintenance of analgesia with the green light lasted for 2 days in CCI mice after the 7-day green light application and extended for 10 days after the 14-day green light treatment (11). This study also revealed that the extended analgesic effect of green light may be related to the duration of exposure.

The precise peripheral and central mechanisms that maintain the analgesic effects of green light remain unclear. As mentioned above, the inhibition of inflammation, protection of neurons from injury, reduced central pain-related cortex activity, enhancement of descending pain inhibitory pathways, and activation of the endogenous opioid system and endocannabinoid system may partly explain the extended analgesic effect of the green light. Significant differences exist in the ability of green light to maintain analgesia among different studies. The variety of neuropathic pain models and patterns of green light may be superficial reasons. The activation of certain circuits in the brain, which rely on different retinal cells, LGN cells, and other cortical areas following specific intensities and durations of green light, may be fundamental. Both peripheral and central samples were collected at the end of green light exposure. In the future, biochemical changes in samples such as serum, CSF, and other brain tissues and in neuronal plasticity should be compared between the end of green light exposure and the point at which the analgesic effect has completely disappeared.

## 6 Clinical trials

Green light can result in a reduction in pain related to a variety of conditions, such as migraine, fibromyalgia, and headache (9, 10, 46, 47). However, its analgesic effect has not been evaluated in patients with clinical neuropathic pain. The safety and efficacy of green light should be studied first in clinical studies. Large-scale real-world assessments need to be designed according to these previous clinical studies. For example, consider the following pilot study. Adult patients with a known diagnosis of neuropathic pain will be recruited. Patients who are colorblind or have serious mental illness will be excluded. To avoid the placebo effect and determine the analgesic effects of a specific color, blank controls and other colors of light, such as white light, will be considered. Patients will be randomly divided into different intervention groups. The primary efficacy outcome for this study may include changes in patient-reported pain scores or analgesic medicine use after intervention. Process events should be tracked via detailed, standardized profiles such as Patient-Reported Outcome Measurement System Profile 57 (PROMIS-57) (47). Notably, spectacles and LED strips have been used to investigate the analgesic effects of green light (9, 10, 48). The intensity of green light can be adjusted over a wider range when using LED strips. Thus, LED strips are recommended first in clinical trials. In the proposed study, the subject was instructed to maintain the 2 m green light strips within his field of view while remaining awake during the therapy and to change the distance of the light source between 1 and 2 m from his eyes. Light intensity between 4 and 100 lux corresponds to the distances described above (9, 10). As shown in Table 2, the analgesic effect of green light (8 h/day) on neuropathic pain has been discovered within 2 weeks (8, 12, 45). The degree of fibromyalgia decreased after 2 weeks of green light therapy (4 h/day) (47). Therefore, green light therapy for 4 h per day for 2 weeks is proposed in the future.

## 7 Conclusions and implications

Numerous animal studies support that green light alleviates neuropathic pain. However, there are many unanswered questions in this area. As shown in Tables 1 and 2, the different patterns of green light treatment had different analgesic effects on and prolonged antinociceptive effects on different types of neuropathic pain. In fact, light features such as intensity and wavelength are detected by different ganglion cells. M-type ganglion cells, which project to the vLGN in the large cell layer, mainly undergo rod convergence and can sense the intensity and movement of light. P-type ganglion cells projecting to the vLGN small cell layer are responsible for color vision and transmit green signals from cones. Different classes of ipRGCs vary in light sensitivity, function, and intracerebral projection modulation (49, 50). In addition, the LGN is divided into a dorsal part (dLGN), a ventral part (vLGN), and an IGL according to the morphology and distribution of nerve terminals (51, 52). Interestingly, different features of light could have different effects on pain modulation following the activation of the same type of neurons of the LGN (Figure 1). For example, red light facilitates the development of neuropathic pain via the activation of GABAergic neurons in the vLGN (7). However, chronic pain was inhibited after the activation of GABAergic neurons in the vLGN/IGL by bright light (38). We posit that different characteristics of green light, such as exposure duration and intensity, may facilitate its transmission to different areas of the LGN through various ganglion cells. Thus, the activation of different retino-vLGN tracts and their projecting cortices may contribute to different effects on neuropathic pain. The DRN and the ACC play central roles in regulating neuropathic pain-related emotional disorders such as anxiety and depression. Thus, the effects of green light therapy on depression and anxiety merit further study. Finally, studies on the clinical application of green light therapy in the treatment of neuropathic pain are limited. The safety and efficacy of green light should be considered first in the clinic. Moreover, the best green light treatment parameters for various neuropathic pain conditions should also be further investigated.
